# Hooked by the tongue: buccal parasitism of *Moenkhausia* spp. (Ostariophysi: Acestrorhamphidae) by *Paracymothoa astyanaxi* (Isopoda: Cymothoidae)

**DOI:** 10.1590/S1984-29612025063

**Published:** 2025-11-03

**Authors:** Artur Firmino, André Vital, Rayssa Nayara, Veronica Slobodian

**Affiliations:** 1 Universidade de Brasília – UnB, Instituto de Ciências Biológicas, Departamento de Zoologia, Brasília, DF, Brasil; 2 Universidade de Brasília – UnB, Programa de Pós-graduação em Zoologia – PGZOO, Laboratório de Ictiologia Sistemática – LIS, Brasília, DF, Brasil; 3 Universidade de Brasília – UnB, Instituto de Ciências Biológicas, Programa de Pós-graduação em Zoologia – PGZOO, Laboratório de Sistemática de Insetos – LASIN, Brasília, DF, Brasil

**Keywords:** Freshwater, Upper River Tocantins, Crustacea, Ectoparasite, host-parasite interaction, Água doce, Alto Rio Tocantins, Crustacea, Ectoparasita, interação hospedeiro-parasita

## Abstract

Parasite-host interactions are shaped by environmental and biological factors, leading to diverse host impacts ranging from tissue damage to physiological impairments. The Tocantins-Araguaia basin, particularly the Upper Rio Tocantins region, harbors remarkable fish diversity and endemism, making it a critical area for parasitological research. Here, we report the first record of parasitism by isopods of the genus *Paracymothoa* in *Moenkhausia* species (*M. aurantia* and *M. goya*) from the Upper Rio Tocantins basin. The parasites (*Paracymothoa astyanaxi*) were located in the buccal cavity and frequently associated with visible lesions, including tongue amputation, a known outcome of their trophic behavior. This finding contributes to the understanding of parasitic diversity associated with *Moenkhausia* and underscores the relevance of parasitological surveys in Neotropical freshwater systems. It also provides insights into local ecological pressures and supports the development of conservation strategies for endemic ichthyofauna in biodiversity-rich and ecologically sensitive areas.

## Resumo

Interações parasito-hospedeiro são influenciadas por fatores ambientais e biológicos, podendo resultar em diversos impactos para o hospedeiro, desde danos teciduais até prejuízos fisiológicos. A bacia do Tocantins-Araguaia, em especial o Alto Rio Tocantins, abriga alta diversidade e endemismo de peixes, tornando-a relevante para estudos parasitológicos. Aqui, registramos pela primeira vez o parasitismo por isópodes do gênero *Paracymothoa* em espécies de *Moenkhausia* (*M. aurantia* e *M. goya*) desta bacia. Os isópodes (*Paracymothoa astyanaxi*) estavam alojados na cavidade bucal, frequentemente associados a lesões visíveis, incluindo amputação da língua, um efeito conhecido do comportamento trófico desses crustáceos. O achado contribui para o conhecimento da diversidade parasitária em *Moenkhausia* e reforça a relevância de levantamentos parasitológicos em sistemas Neotropicais de água doce. Além disso, fornece *insights* sobre pressões ecológicas locais para fomentar estratégias de conservação da ictiofauna endêmica em áreas biodiversas e ecologicamente sensíveis.

Parasites can negatively affect their hosts through multiple mechanisms, including tissue damage and physiological impairments (Yamano et al., 2011). Parasitism rates are modulated by both host traits and environmental conditions (Arostegui et al., 2018). Habitat variation within ecosystems directly influences host-parasite dynamics by altering host distribution patterns and potentially restructuring food-web interactions (Smith, 2001). This complex interplay of biotic and abiotic factors not only governs parasitic relationships but also contributes to the unique biodiversity observed in the Tocantins-Araguaia basin.

The Tocantins-Araguaia drainage represents the largest river system entirely contained within Brazilian territory (Dagosta & Pinna, 2019). Originating in the Central Plateau's Cerrado biome, its headwaters serve as a critical hydrological resource. The system's biogeographic complexity permits division into three distinct regions: Upper Tocantins, Lower Tocantins, and Araguaia (Dagosta & Pinna, 2019). Recognized as a hotspot of aquatic endemism, this ecoregion faces escalating anthropogenic threats, with the Upper Tocantins basin exhibiting particularly high fish diversity and endemism (91 species; Chamon et al., 2022).

Among this rich ichthyofauna, Characiformes emerge as a dominant component, representing one of the most diverse and widespread Neotropical fish groups (Fricke et al., 2025). Within this order, *Moenkhausia* Eigenmann, 1903 comprises approximately 84 valid species distributed across South American drainages (Fricke et al., 2025). Although the species currently assigned to *Moenkhausia* do not form a monophyletic group (e.g., Melo et al., 2024), the genus is usually diagnosed by an exclusive combination of traits, including: biserial premaxillary teeth, pentacuspid maxillary teeth, complete lateral line with perforated scales, scaled caudal fins, and 11 dorsal-fin rays (ii+9) (Eigenmann & Myers, 1917). Their vibrant coloration and peaceful demeanor make *Moenkhausia* species commercially valuable in the ornamental trade (Marinho & Dagosta, 2023). Also, most species exhibit restricted distributions, being endemic to single basins or drainages (e.g., Marinho & Dagosta, 2023), rendering them particularly vulnerable to local environmental conditions. This microendemism influences ecological adaptations, including dietary specialization (Silva & Hahn, 2009; Tófoli et al., 2010; Caldatto et al., 2023) and parasite susceptibility.

**Figure 1:**
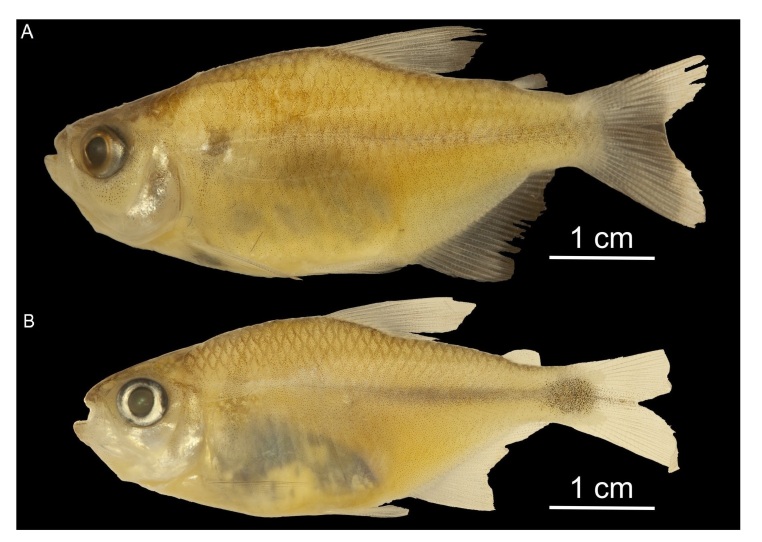
Analyzed specimens of (A) *Moenkhausia aurantia* (CIUnB 2064); (B) *Moenkhausia goya* (CIUnB 2143).

Parasitism in *Moenkhausia* remains poorly documented, with reports limited to *M. intermedia* Eigenmann 1908 and *M. costae* (Steindachner 1907) in the Jaguaribe River basin (Falkenberg et al., 2024). Among 18 parasite taxa recorded in this basin, only two represent crustaceans such as Ergasilidae and Lernaeidae, (Falkenberg et al., 2024). Notably, no parasitological studies exist for *Moenkhausia* species in the Tocantins-Araguaia system, and only one register of a crustacean fish parasite is known for the system (Michelan et al., 2023). Here, we report the first cases of parasitism in *Moenkhausia aurantia* Bertaco, Jerep & Carvalho 2011, a species with a relatively broad distribution across the Upper Tocantins and São Francisco basins (in the states of Goiás, Minas Gerais, and the Federal District, Brazil), and *Moenkhausia goya* Deprá Azevedo-Santos, Vitorino Júnior, Dagosta, Marinho & Benine 2018, a species endemic to the Upper Tocantins (Goiás and Federal District) (Figure 1). Although *Paracymothoa* parasitism has been documented in other Neotropical fish species, this represents the first record for both *Moenkhausia aurantia* and *M. goya*, as well as the first reported case for any *Moenkhausia* species in the Upper Tocantins basin, underscoring significant gaps in our understanding of regional host-parasite dynamics.

Fish were collected between November 2022 and January 2024 using dip nets and seine nets. Specimens were euthanized with eugenol, fixed in formalin, and preserved in 70% ethanol. Hosts are deposited in the Ichthyological Collection of the University of Brasília (CIUnB), and parasites are cataloged in the Aquatic Invertebrates Collection (CIAq: CIAaq-80001). Ectoparasites were screened following Eiras et al. (2000), with detailed examination of the oral and branchial cavities. Hosts were identified using taxonomic keys and original species descriptions; parasites were identified based on Lemos de Castro & Loyola-Silva (1985) and Luque et al. (2013).

**Figure 2:**
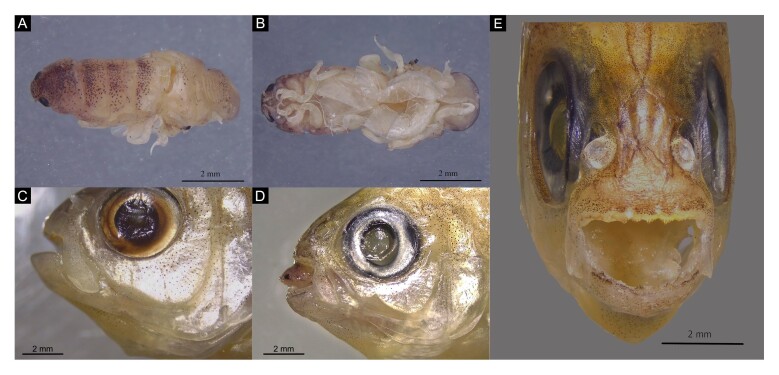
Examined specimen of *Paracymothoa astyanaxi* and lesions caused by the parasite. (A) Dorsal view of *Paracymothoa astyanaxi* (CIAq-80001); (B) Ventral view; (C) Prominent ventral region of oral cavity in *Moenkhausia goya* (CIUnB 2143); (D) Presence of a *Paracymothoa astyanaxi* individual in oral cavity of *Moenkhausia aurantia* (CIUnB 2064); (E) Damage by tongue amputation of *Moenkhausia aurantia* (CIUnB 2064).

**Figure 3:**
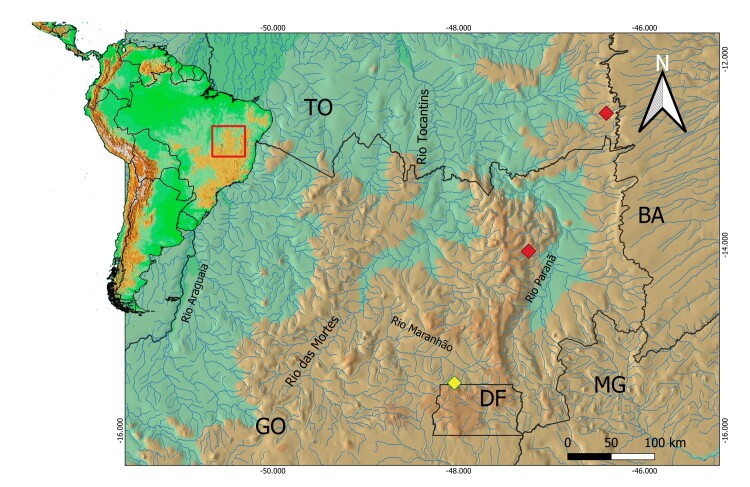
Sampling sites of recorded parasitism events in the Upper Tocantins. Red diamond- *Moenkhausia aurantia*; Yellow diamond - *Moenkhausia goya*. Black lines: division of South America countries and Brazilian States (abbreviations). Main rivers indicated.

We documented two *M. aurantia* specimens parasitized by *Paracymothoa astyanaxi* Lemos de Castro, 1955 (Figure 2A-B). One individual from Aurora do Tocantins (GO) hosted a buccal isopod (Figure 2C); another from Alto Paraíso de Goiás (GO) showed characteristic lesions. A *M. goya* specimen from Brasília (DF) exhibited complete tongue loss (Figure 2D), a pathognomonic sign of *Paracymothoa* spp, which was also observed in *M. aurantia* (Figure 2E). All affected fish displayed ventral oral protrusions, suggesting consistent parasite-induced pathology. These records demonstrate *P. astyanaxi* broad distribution in the Upper Tocantins (Figure 3). A total of 158 *M. aurantia* and six *M. goya* specimens were examined. In *M. aurantia,* the prevalence was 1.27% (2/158), with a mean intensity of 1.0 and mean abundance of 0.013. In *M. goya*, the prevalence was 16.7% (1/6), with a mean intensity of 1.0 and a mean abundance of 0.167 (see Material Examined).

The Cymothoidae family comprises approximately 380 species of parasitic isopods (Ahyong et al., 2011), predominantly marine, but with freshwater representatives in South America, Africa, and Asia. Tropical regions harbor their peak diversity, and all species in the family are obligate parasites of fish (Smith et al., 2014). Cymothoids exhibit site specificity, attaching to oral cavities, gills, or perforating host tissues (Lemos de Castro & Loyola-Silva, 1985), often causing severe mechanical damage. In addition to these mechanical impacts, representatives of the family Cymothoidae also cause significant physiological changes in the host fish. Such damage results from the action of the pereopods (prehensile appendages), which compress the tongue region causing its atrophy. The parasite thus assumes a replacement trophic function, feeding directly on material ingested by the host. This condition compromises not only fish feeding and respiration, but can also increase its vulnerability to predation, especially in small-sized species (Abilhoa, 2007; Gomiero et al., 2012).

This first record of *P. astyanaxi* parasitizing *Moenkhausia* in the Upper Tocantins underscores the need to investigate parasitic interactions in biodiverse ecosystems. Documentation is particularly crucial for aquarium trade species, since South American tetras are globally traded (Geller et al., 2020), with wild-caught specimens often harboring parasites (Plaul et al., 2018). Also, Neotropical freshwater systems host rich crustacean parasite diversity (e.g., Oliveira et al., 2024), warranting comprehensive study.

This study provides insights into the parasite fauna of Neotropical freshwater fishes, particularly for *Moenkhausia* species in the Upper Tocantins basin. Documenting these host-parasite interactions is essential for advancing our understanding of coevolutionary dynamics and ecological networks in freshwater ecosystems. Moreover, these results carry conservation implications, as anthropogenic pressures continue to threaten aquatic biodiversity. The observed pathological effects of parasitic crustaceans on host fitness (Tavares-Dias et al., 2014) underscore their potential role as bioindicators of ecosystem health and regulators of fish community structure. Our findings emphasize the urgent need to integrate parasitological data into conservation planning for the Tocantins-Araguaia basin and other Neotropical freshwater systems facing similar environmental challenges.

## Examined Material

*Moenkhausia aurantia*- All from Brazil. CIUnB 2144, 1 examined, 36,25mm SL, Tocantins, Aurora do Tocantins municipality, rio Tocantins basin, Rio Monteiro, 12°34'50.5”S 46°24'37.2”W; CIUnB 2064, 157 examined, 44,32mm-25,58mm SL, Goiás, Alto Paraíso de Goiás municipality, rio Tocantins basin, Riacho Indaiá (Antropizado), 14°03'59.71”S 47°14'49.37”W.
*Moenkhausia goya*- All from Brazil. CIUnB 2143, 1 examined, 45,75mm SL, Brazil, Distrito Federal, Brasília municipality, rio Tocantins basin, Rio Palma, 15°29' 02.4”S 48°02' 42.0”W. CIUnB 1865, 2 examined, 39.47-46.50mm SL, Brazil, Distrito Federal, Brasília municipality, rio Tocantins basin, Córrego do Ouro, 15 30 41.88”S 47 55 47.96”W. CIUnB 2278, 1 examined, 41.21mm SL, Brazil, Distrito Federal, Brasília municipality, rio Tocantins basin, Riacho Palmeira, 15°31'44.3”S 47°44'27.1”W. CIUnB 378, 2 examined, 32.86-34.06mm SL, Brazil, Distrito Federal, Brasília municipality, rio Tocantins basin, Rio do Sal, 15°30'17.0”S 48°10'36.6”W.
*Paracymothoa astyanaxi*- All from Brazil. CIAq-80001, 1 examined, Tocantins, Aurora do Tocantins municipality, rio Tocantins basin, Rio Monteiro, 12°34'50.5”S 46°24'37.2”W

## Data Availability

Data will be made available on request.
